# Estimating infection prevalence: Best practices and their theoretical underpinnings

**DOI:** 10.1002/ece3.4179

**Published:** 2018-06-12

**Authors:** Ian F. Miller, India Schneider‐Crease, Charles L. Nunn, Michael P. Muehlenbein

**Affiliations:** ^1^ Department of Ecology and Evolutionary Biology Princeton University Princeton New Jersey; ^2^ Department of Evolutionary Anthropology Duke University Durham North Carolina; ^3^ Department of Anthropology Stony Brook University Stony Brook New York; ^4^ Department of Psychology University of Washington Seattle Washington; ^5^ Duke Global Health Institute Duke University Durham North Carolina; ^6^ Department of Anthropology Baylor University Waco Texas

**Keywords:** epidemiology, helminth, methods, prevalence, primate

## Abstract

Accurately estimating infection prevalence is fundamental to the study of population health, disease dynamics, and infection risk factors. Prevalence is estimated as the proportion of infected individuals (“individual‐based estimation”), but is also estimated as the proportion of samples in which evidence of infection is detected (“anonymous estimation”). The latter method is often used when researchers lack information on individual host identity, which can occur during noninvasive sampling of wild populations or when the individual that produced a fecal sample is unknown. The goal of this study was to investigate biases in individual‐based versus anonymous prevalence estimation theoretically and to test whether mathematically derived predictions are evident in a comparative dataset of gastrointestinal helminth infections in nonhuman primates. Using a mathematical model, we predict that anonymous estimates of prevalence will be lower than individual‐based estimates when (a) samples from infected individuals do not always contain evidence of infection and/or (b) when false negatives occur. The mathematical model further predicts that no difference in bias should exist between anonymous estimation and individual‐based estimation when one sample is collected from each individual. Using data on helminth parasites of primates, we find that anonymous estimates of prevalence are significantly and substantially (12.17%) lower than individual‐based estimates of prevalence. We also observed that individual‐based estimates of prevalence from studies employing single sampling are on average 6.4% higher than anonymous estimates, suggesting a bias toward sampling infected individuals. We recommend that researchers use individual‐based study designs with repeated sampling of individuals to obtain the most accurate estimate of infection prevalence. Moreover, to ensure accurate interpretation of their results and to allow for prevalence estimates to be compared among studies, it is essential that authors explicitly describe their sampling designs and prevalence calculations in publications.

## 1 INTRODUCTION

Prevalence, a key measure in studies of disease ecology, is defined as the percentage of individuals in a population infected with a given pathogen (Jovani & Tella, 2006). This measure describes the occurrence of a pathogen in a population and is an essential component of mathematical models in epidemiology (Kermack & McKendrick, 1927). Because determining the “true” prevalence of a pathogen in a population would require exhaustive sampling from every individual in the target population, studies generally estimate pathogen prevalence by determining the infection status of a proportion of the population via necropsy or sampling of feces, urine, blood, or saliva (Jovani & Tella, 2006). Because invasive procedures may be impractical or prohibited, particularly in studies of threatened populations, the analysis of noninvasive samples of material that potentially contains evidence of infection (e.g., feces or urine) is often preferred (Leendertz et al. 2006).

**Figure 1:**
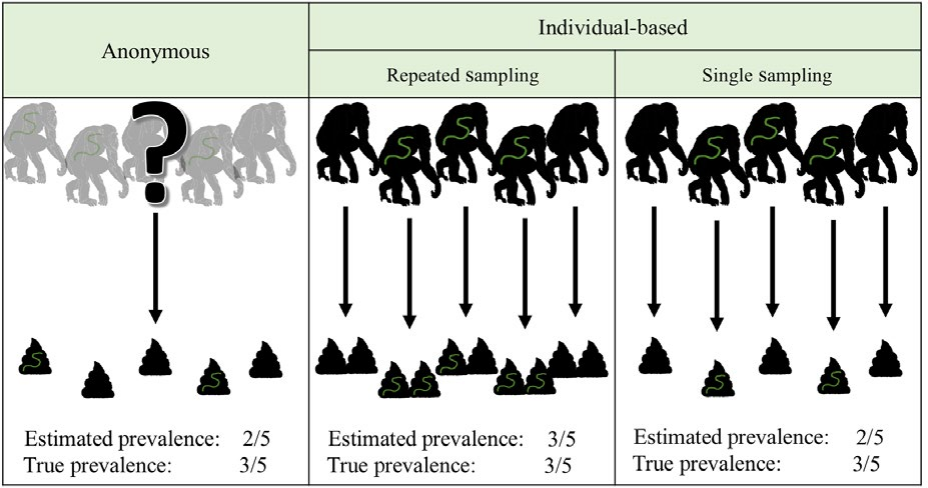
Prevalence estimation methods. In anonymous prevalence estimation, the origin of samples is unknown, and any information about the number of hosts that generated the samples cannot be used in estimating prevalence. In individual‐based prevalence estimation with single sampling, each sample is paired to a different host. In individual‐based prevalence estimation with repeated sampling, multiple samples are paired to each host, enabling more accurate estimates of prevalence when infected hosts do not always produce samples containing evidence of infection

Methods for estimating prevalence from such samples can be placed in two categories (Figure 1). “Individual‐based estimations” are made when samples are collected from known individuals. Multiple samples may be collected from each individual, and prevalence is estimated as the proportion of individuals in which at least one sample contains evidence of infection. “Anonymous estimations” are made when samples are collected from the environment without being matched to the individual from which they originated, with prevalence estimated as the proportion of samples containing the evidence of infection. A study that reports prevalence as a proportion of infected samples employs anonymous estimation even if the number of sampled individuals or size of the sampled group is given, unless the number of samples is equal to the number of sampled individuals, in which case we classify the estimation method as individual‐based with single sampling.

Several past studies have discussed the accuracy of prevalence estimation methods. Muehlenbein (2005) found that the prevalence of multiple helminth species increased as *Pan troglodytes schweinfurthii* individuals were sampled repeatedly, and recommended that all researchers should standardize their prevalence estimation methods by sampling individuals repeatedly and only using individual‐based prevalence estimation methods. Huffman, Gotoh, Turner, Hamai, and Yoshida (1997) asserted that anonymous estimation methods are biased relative to individual‐based methods, but provided only empirical evidence from a single population of *P. troglodytes schweinfurthii* to back this claim. Several other authors (including Murray, Stem, Boudreau, & Goodall, 2000; Gillespie, 2006; Muehlenbein, Schwartz, & Richard, 2003) have cautioned against anonymous estimation methods or claimed to have benefited from individual‐based estimation methods, but the comparative performance of the two methods has yet to be rigorously examined mathematically or empirically.

Here, we formally compare the performance of individual‐based and anonymous prevalence estimation methods. We begin by presenting a simple mathematical model that demonstrates the differences in bias between the two. Our model guides us toward two specific predictions, described below, which we investigate with empirical data on gastrointestinal helminth infections of primates taken from the Global Mammal Parasite Database (GMPD) (Nunn & Altizer, 2005; Stephens et al., 2017). We focus on these hosts and parasites because helminths are the main parasite for which fecal sampling occurs, and sampling challenges are common in primates due to their complex ecology and some species' threatened status.

## 2 THEORETICAL EXAMINATION OF BIASES IN PREVALENCE ESTIMATION

### 2.1 Individual‐based prevalence estimation

Using the definition above, “true” prevalence (*P*) is defined mathematically as:

(1) $$P=\frac{I}{N}$$

In this equation, *I* is the number of infected individuals in a population, and *N* is the total number of individuals. True prevalence is a theoretical representation of the actual occurrence of a pathogen in a discrete population.

In practice, the true prevalence of a pathogen is often impossible or impractical to measure, and sampling designs are restricted to providing an estimate of prevalence ($\hat{P}$). An estimate of prevalence is biased if its expected value ($E[\hat{P}]$) is not equal to the true prevalence (*P*). If $E[\hat{P}]$ < *P*, prevalence will be underestimated, while if $E[\hat{P}]$ > *P*, prevalence will be overestimated.

In individual‐based methods, $\hat{P}$ is calculated by dividing the number of individuals observed to be infected (*i*) by the total number of individuals that were sampled (*n*):

(2) $$\hat{P}=\frac{i}{n}$$

When *n *< *N,* this calculation assumes that sampling is random. Using Equation (3), we can calculate the expected value of $\hat{P}$ while incorporating information about repeated sampling of individuals and the efficacy of the method used to detect evidence of infection in a sample.

(3) $$E\left[\hat{P}\right]=P\left(1-{\left(1-D\right)}^{X}\right)$$

In this equation, *D* is the probability that a sample containing evidence of infection is detected as such (i.e., detection rate), and *X* is the number of samples collected from each individual (see Appendix for derivations of all equations). If *D *=* *1, the expected value of $\hat{P}$ is equal to *P*, and thus, $\hat{P}$ is an unbiased estimator of prevalence. If *D *<* *1, $\hat{P}$ is a negatively biased estimator of *P*. Thus, $\hat{P}$ underestimates *P* whenever the probability of a false negative is greater than zero. Many factors can cause the detection rate to fall below 1. For example, certain chemicals used in the past are not conducive to long‐term preservation of delicate specimens, such as some protozoa. Many protozoa and even some of the more common helminths can also be difficult to distinguish from fecal debris. However, bias due to false negatives decreases as per‐individual sampling effort (*X*) increases, because 1 ‐ (1 ‐ *D*)^*X*^ approaches 1 as *X* increases.

Further bias is introduced if there is variation in the presence of evidence of infection in samples from an infected individual. For example, egg production by helminths can vary with age of the parasite population and the presence of co‐infections by other parasites (Muehlenbein & Lewis, 2013). In Equation (4), we define *F* as the proportion of an infected individual's samples that contain evidence of infection, or the occurrence rate. In this scenario, the expected value of $\hat{P}$ is as follows:

(4) $$E\left[\hat{P}\right]=P\left(1-{\left(1-FD\right)}^{X}\right)$$

If *D *<* *1 or *F *<* *1 in this equation, $\hat{P}$ is a negatively biased estimator of *P*, regardless of the sampling effort. However, the bias still decreases as *X* increases because repeatedly sampling individuals increase the likelihood that infected individuals will be correctly identified as such. It is not necessary to distinguish between the effects of false negatives and variation in the presence of evidence of infection in order to infer the presence of bias, because the *F* and *D* terms are multiplicatively combined.

Muehlenbein (2005) provides an empirical example of how repeated sampling can mitigate the bias introduced when not all samples from infected individuals test positive. He found that within a population of wild chimpanzees, cumulative parasite richness (number of unique intestinal parasites infecting a given host) significantly increased for every sequential sample (up to four samples) taken per animal. In the same study, the most commonly occurring parasites were found in all of the serial samples of only a fraction of the chimpanzees, and not one of the twelve parasitic species recovered from the group was found in all samples from any one animal.

Sampling protocols that only collect one or a few samples per individual are particularly prone to large biases in prevalence estimation, especially when *D* and or *F* are much less than 1. To observe these biases, many estimates of prevalence from multiple studies of the same disease system would have to be compared. In a dataset of many disease systems that vary significantly in terms of *P*,* F*, and *D*, the complex interaction between these variables would obscure the pattern of how increased sampling effort corresponds to increased estimated prevalence.

### 2.2 Anonymous prevalence estimation

In anonymous estimation methods, prevalence is estimated by dividing the number of samples that test positive (*S*_I_) for the pathogen by the total number of samples collected (*S*_N_) (Equation (5)). This approach is based on the assumption that the proportion of infected samples reflects the proportion of infected individuals in the population:

(5) $$\hat{P}=\frac{S_{I}}{S_{N}}$$

Note that measures of population size are not present in the equation. A major assumption underlying this calculation is that sampling is random. The expected value of $\hat{P}$ for anonymous sampling is:

(6) $$E[\hat{P}]=\text{PFD}$$

The expected value of prevalence is the same for anonymous estimations of prevalence and individual‐based estimations of prevalence from studies in which individuals are only sampled once (i.e., Equation (4) reduces to Equation (6) when *X *=* *1). In all other cases, assuming that the detection rate (*D*) and occurrence rate (*F*) are less than 1, the bias for anonymous prevalence estimation is more negative than the bias for individual‐based prevalence estimation (see Appendix). This effect arises because anonymous estimation is unable to account for infected individuals producing samples that do not contain any evidence of infection. Individual‐based estimation methods can partially overcome this problem by accounting for the repeated sampling of individuals.

A sensitivity analysis of the effect of the values of *P*,* F*,* D*, and *X* on the difference in bias between individual‐based and anonymous prevalence estimation methods is given in the Appendix The key finding that emerges from this analysis is that the difference between the prevalence estimates generated using the two methods increases proportionally to *P* and is greater for higher values of *X*. Individual‐based estimates of prevalence are greater than or equal to anonymous estimates of prevalence for all values of all parameters.

### 2.3 Predictions

Our theoretical treatment of prevalence estimation gives rise to two predictions with regard to the performance of individual‐based and anonymous estimations of prevalence. First, individual‐based estimates of prevalence from studies in which individuals are repeatedly sampled should be on average higher than anonymous estimates of prevalence, assuming that random sampling of individuals or samples occurred in all studies. This prediction arises because we expect that less than 100% of samples from infected individuals will show evidence of infection (i.e., *F* and/or *D *<* *1), based on technical and biological failures to detect infections as described above. Second, we predict equivalence between individual‐based estimates of prevalence from studies with single sampling of individuals and anonymous estimates of prevalence. If differences in sampling bias toward infected individuals exist between these two categories of prevalence estimates, then these estimates of prevalence will differ, based on the equations and assumptions given above (Equations (4) and (6)). To test both predictions, the estimates of prevalence being compared must represent a random sample of parasites, hosts, and laboratory techniques, as this helps account for variation in *F* and *D* among studies.

## 3 EMPIRICAL ASSESSMENT OF SAMPLING DESIGN PERFORMANCE

### 3.1 Methods

We evaluate the above predictions using empirical data on gastrointestinal helminth parasite infections in primate hosts, detected through fecal sampling, from the GMPD (Nunn & Altizer, 2005; Stephens et al., 2017), a database compiled through systematic literature searches for infectious diseases of primates. The data we extracted span 31 host genera and 64 parasite genera, and are drawn from 123 published papers representing multiple different laboratories and authors. Thus, we view our dataset as a random sample of prevalence estimates. We extracted the prevalence estimates, sample sizes, host species, and parasite species from each relevant entry in the GMPD and then coded the prevalence estimates as either “individual‐based” or “anonymous.” All ambiguously described prevalence estimates were coded as anonymous. We did not extract anonymous estimates of prevalence from studies where the number of individuals sampled was stated and equal to the number of samples, but instead considered these data to represent individual‐based estimates of prevalence with single sampling. When a study reported separate prevalence estimates for age and sex classes within a population, or when a study reported prevalence estimates for study subpopulations (i.e., different social groups within a park), we pooled the data and calculated a combined prevalence estimate. This was carried out to make these data consistent with data from other studies that pooled data across demographic groups and subpopulations. Prevalence estimates reported for the same study population in different years were treated as separate data points, because such studies often investigated changes in prevalence over time due to factors such as environmental change. In several cases, multiple prevalence estimates corresponding to different laboratory techniques were given for a host–parasite pair within a study and were treated as separate data points. Finally, we removed entries for which all forms of estimated prevalence were equal to 0, which indicates that the authors searched for the parasite but failed to find it.

To test our first prediction, we first compared anonymous and individual‐based prevalence estimates from studies that provide both of these types of estimates using a paired *t*‐test. We then conducted a statistical analysis to assess differences between individual‐based and anonymous estimates of prevalence in the entire dataset. In this larger analysis of all available data, we performed model selection with Akaike information criterion (AICc), which selects an optimum model based on maximum likelihood (Akaike, 1998), in the R statistical platform (R Core Team 2014) using the “MuMin” (Barton, 2009) and “lme4” (Bates, Maechler, Bolker, & Walker, 2014) packages. We averaged models that were within 10 AIC units of the best model. All candidate models were linear. We considered all combinations of prevalence estimation type (“individual” or “anonymous”) and the interaction between host genus and parasite genus as predictor variables of prevalence estimate. We included random effects of host genus and parasite genus in all candidate models. We did not include interactions between prevalence estimation type and host or parasite genus, as no effects were predicted and doing so would not help in evaluating our specific predictions about estimation type and prevalence. We chose not to use models that specifically incorporate phylogenetic effects (such as phylogenetic generalized least squares, i.e., PGLS), because such models cannot incorporate more than one data point per species, which would prevent the comparison of estimates of prevalence of different parasites from the same host. Additionally, such methods would control for the phylogeny of either the host or the parasite, but not both. However, we were able to control for some phylogenetic effects by including host and parasite genus as random effects in all candidate models.

To test our second prediction, we investigated differences between individual‐based prevalence estimates from studies that sampled individuals only once and anonymous prevalence estimates using the model selection approach described above.

### 3.2 Results

In total, we extracted 737 total entries on helminth infection prevalence estimated through fecal sampling from the GMPD. Of these, 425 give an individual‐based estimate of prevalence, 349 give an anonymous estimate of prevalence, and 37 give both individual‐based and anonymous estimates of prevalence. Our data span 31 host genera and 64 parasite genera. Further details are provided in Table S1.

**Figure 2:**
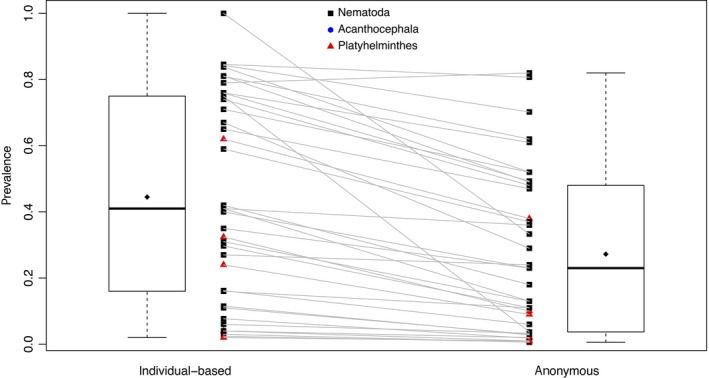
Paired individual and anonymous prevalence estimates. Data shown are individual‐based and anonymous prevalence estimates calculated for the same host–parasite pair within a study. Lines connect paired prevalence estimates. Colors indicate the phylum of the parasite. Diamonds within the boxplots show mean values

Among the 37 entries that provided both individual and anonymous estimates of prevalence, we find that individual sampling led to higher estimates of prevalence (Figure 2). The mean of the individual‐based estimates of prevalence is 44.5% (*SD* = 30.9%), and the mean of the anonymous estimates of prevalence is 27.2% (*SD* = 24.7%), leading to a mean difference of 17.3% (95% CI: 11.8%–22.7%). Only one entry reports a higher anonymous than individual‐based prevalence estimate, and in that case, the difference is very small (3%). In support of Prediction 1, a paired *t*‐test reveals that anonymous estimates of prevalence are significantly lower than individual‐based estimates of prevalence (*t*_36 _= 6.46, *p *<* *0.0001).

**Figure 3:**
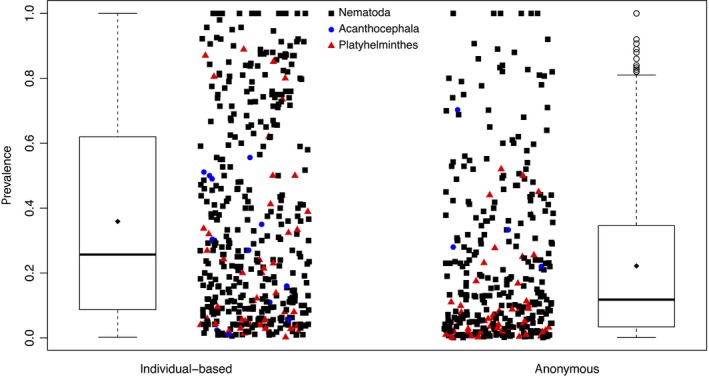
Individual‐based and anonymous prevalence estimates. Data shown are all measures of individual and anonymous prevalence extracted from the GMPD (includes all data shown in Figure 2). Colors indicate the phylum of the parasite. Diamonds within the boxplots show mean values

We observe this same pattern in the broader analysis of the full dataset of prevalence estimates from studies that reported one or both types of estimates. The results of the model selection process reveal that the top model received 100% of the weight (Table 1). Prevalence estimation type has a relative importance score of 1. In this model, anonymous prevalence estimation is again associated with substantially decreased prevalence (coefficient = −0.1217, *t* = −5.87, *p *<* *0.0001). Thus, measures of prevalence from individual‐based designs are on average 12.17% higher than those from anonymous designs after accounting for the genus of the host and the parasite (Figure 3), also supporting our first prediction.

**Table 1:** Multimodel inference of the effect of individual‐based vs. anonymous prevalence estimation method

Prevalence estimation method	Host genus by parasite genus interaction	Intercept	*df*	Log(lik)	AICc	ΔAICc	Weight
+		0.32	5	−105.17	220.4	0	1

Note

AICc: Akaike information criterion.

Table 1 shows the top model selected for the analysis of prevalence. “+” symbols indicate included variables. All other models had ΔAICc > 10, were not included in the averaged model, and are not shown.

**Figure 4:**
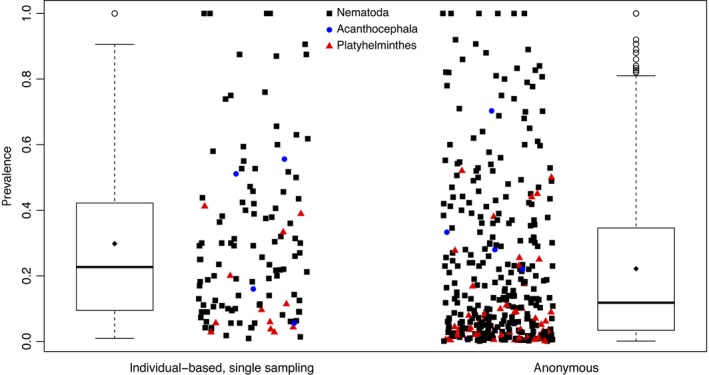
Individual‐based estimates of prevalence using single sampling and anonymous estimates of prevalence. Data shown are individual‐based estimates of prevalence taken from studies that sampled individuals only once, and anonymous estimates of prevalence. Colors indicate the phylum of the parasite. Diamonds within the boxplots show mean values

In testing Prediction 2, we find that individual‐based estimates of prevalence from studies with single sampling of individuals (*N *=* *120) differ from anonymous measures of prevalence (Figure 4). Prevalence estimation method has a relative importance score of 0.29 (Table 2). In the averaged model, the coefficient of anonymous prevalence estimation is −0.064 (*Z *=* *2.40, *p *<* *0.02). This indicates that after controlling for other factors, anonymous estimations of prevalence are on average 6.4% lower than individual‐based estimates of prevalence.

**Table 2:** Multimodel inference of the effect of individual‐based prevalence estimation without repeat sampling of individuals vs. anonymous prevalence estimation

Prevalence estimation method	Host genus by parasite genus interaction	Intercept	*df*	Log(lik)	AICc	ΔAICc	Weight
		0.23	4	−7.79	23.7	0	0.71
+		0.27	5	−7.66	25.5	1.79	0.29

Note

AICc: Akaike information criterion.

Table 2 shows the top models selected for the analysis of prevalence. “+” symbols indicate included variables. All other models had ΔAICc > 10, were not included in the averaged model, and are not shown.

## 4 DISCUSSION

Our theoretical examination of prevalence estimation indicates that individual‐based and anonymous methods both underestimate true prevalence (in the likely scenario where either the occurrence rate or detection rate is less than one), yet individual‐based estimates have less negative bias than anonymous estimates. In other words, our equations suggest that individual‐based estimates of prevalence should be larger than anonymous‐based estimates. This effect emerges because individual‐based estimates can make use of information about repeated sampling of individuals, improving the likelihood of identifying infected individuals.

Based on this prediction, we investigated whether the theoretical advantage of individual‐based estimation is reflected in an empirical dataset, finding that, for gastrointestinal helminth parasites of primates detected through fecal samples, individual‐based prevalence estimates are indeed higher than anonymous prevalence estimates. This is true both within studies that provided both individual and anonymous prevalence estimates, and among all studies reporting either one or both estimates of prevalence. These results lend theoretical and empirical support to the long‐held recommendations to use individual‐based sampling and avoid anonymous sampling (Huffman et al., 1997; Muehlenbein, 2005).

Individual‐based estimates of prevalence are expected to be closer to true prevalence than anonymous estimates because they are able to use information about the repeated sampling of individuals; thus, individual‐based estimates from studies in which each host is sampled only once should lose their advantage over anonymous estimates. However, our empirical analysis shows that individual‐based estimates of prevalence from studies in which individuals were sampled only once continue to be on average higher (or relatively positively biased) compared to anonymous estimates of prevalence, after accounting for other factors. This positive relative bias suggests that studies that sample individuals only once and use individual‐based prevalence estimation methods are more biased toward sampling infected individuals than studies employing anonymous prevalence estimation. Because the values of true prevalence are unknown, we cannot determine whether this relative positive bias from nonrandom sampling results in an absolute positive bias.

Sampling infected individuals at a higher rate than uninfected individuals could be the product of unconscious bias of field workers toward preferentially sampling individuals that show physical signs of infection, such as diarrhea or poor body condition. Such individuals might simply be easier or more interesting to follow. Direct observation of individuals almost always occurs in studies using individual‐based estimation methods, but could also occur in studies using anonymous estimation methods in the rare case that individuals are observed but not tracked. Indeed, in our dataset, the number of individuals from which samples were collected was not given for 265 of 349 anonymous estimates of prevalence. Researchers should be cognizant of potential biases toward sampling infected individuals and take special care to obtain samples appropriately when attempting to sample each individual only one time. We note, however, that the preferred strategy should be to always obtain repeated samples from known individuals and that the probability of correctly identifying an individual's infection status increases with the number of repeated samples.

Individual‐based prevalence estimation methods should be used whenever possible. The only way to reduce the bias inherent to anonymous estimation methods is to increase the detection rate (*D*). However, if the methods used to increase the detection rate result in false positives, additional biases may arise. Furthermore, individual‐based estimation methods will always outperform anonymous estimation methods regardless of the detection rate. Even when observations of individuals are impossible, samples collected can be matched to hosts through genetic methods that obtain host DNA in the fecal sample and then allocate the fecal samples to distinct individuals, thus allowing for individual‐based prevalence estimation. This approach was used by Liu et al. (2010) in a study of *Plasmodium* in *Gorilla gorilla* and *P. troglodytes*. If more widely adopted, identifying samples to individuals through similar genetic methods would allow for more accurate prevalence estimates by virtue of individual‐based estimation, while minimizing the disturbance of threatened populations and sampling bias.

Our results rest on several key assumptions. Our theoretical predictions carry the assumption that the proportion of samples containing evidence of infection in the pool of all samples reflects the proportion of infected individuals in the host population. Some parasites (especially those that cause diarrhea) may cause infected individuals to defecate more frequently than uninfected individuals, so some anonymous estimates of prevalence in our empirical dataset may contain a positive bias. This bias would make the differences between anonymous and individual‐based prevalence estimation methods appear smaller, resulting in an underrepresentation of the true differences between the estimation methods. However, other pathogens may cause chronically infected individuals to experience decreased appetite, and thus ingest less food and defecate less frequently. This could give some anonymous estimates of prevalence in our empirical dataset a negative bias, resulting in an over representation of the true differences between estimation methods. Additionally, our theoretical predictions rest on the assumption that sampling is random in all cases. Our comparison between anonymous prevalence estimates and individual‐based prevalence estimates from studies with single sampling suggests that this may not always be the case in practice.

Furthermore, we make several important simplifications during our theoretical treatment of prevalence estimation. We do not incorporate false positives. False positives are less likely to occur in fecal sample analyses that focus on helminth egg detection (relative to analyses that seek to identify larva and protozoa), because fecal debris and or other materials are unlikely to be confused for helminth eggs. However, false negatives do remain an issue, especially in cases where it is difficult to discern helminth eggs from fecal debris and other material in the sample, and in genetic procedures such as PCR (Borst, Box, & Fluit, 2004). We also do not consider parasite misidentification. While this may occur, it would not affect any estimate of prevalence as long as the misidentification is consistent, and parasites of separate species are not identified as members of the same species.

Our empirical analyses may have been affected by discrepancies between our data coding and the actual methods employed in the original studies, as many papers from which we collected empirical data were unclear in their descriptions of sample collection and prevalence estimation. However, we classified all ambiguously described methods as anonymous estimation, so any incorrect classifications were almost certainly individual‐based estimated being classified as anonymous estimates. This would obscure differences between estimation types, making it a conservative practice.

In conclusion, we demonstrate theoretically that estimating prevalence as a proportion of infected individuals (individual‐based estimation), rather than as a proportion of samples containing evidence of infection (anonymous estimation), gives a higher and less negatively biased estimate of true prevalence. We found evidence of this pattern in an empirical dataset of gastrointestinal helminth infections of primates. Therefore, repeatedly sampling known individuals should always be the preferred method in parasitological surveys. Because different prevalence estimation methods perform differently, explicit calculations must be published along with prevalence estimates, particularly in studies where the number of samples collected is not equal to the number of individuals sampled. Our results also suggest that nonrandom sampling of individuals may be common in primate parasitology. Therefore, researchers should take care to sample randomly, use methods designed to reduce unconscious sampling bias, and fully and unambiguously report their sampling procedures.

## CONFLICT OF INTEREST

The authors have no conflict of interests to declare.

## AUTHOR CONTRIBUTIONS

IFM compiled data, constructed the mathematical model, designed and conducted statistical analyses, and drafted the manuscript. ISC helped to compile data and design statistical analyses, and drafted the manuscript. CLN and MPM helped conceive of the study and helped draft the manuscript. All authors gave final approval for publication.

## DATA ACCESSIBILITY

The dataset used in this study is available from the Dryad Digital Repository: https://doi.org/10.5061/dryad.51t5s6b.

## Figures and Tables

**Figure A1:**
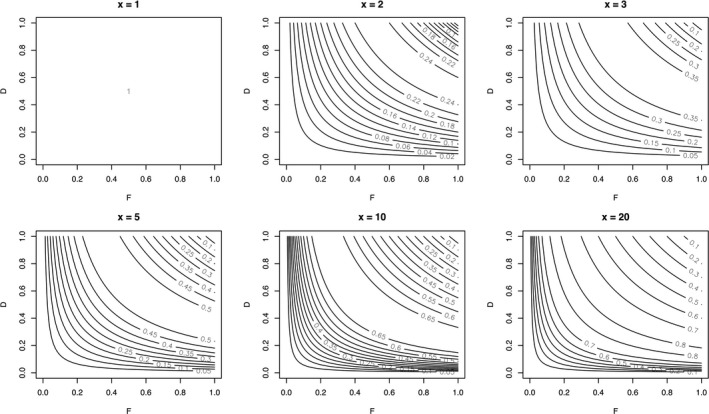
Difference in biases of individual‐based and anonymous prevalence estimation methods. Contour plots show the values of (1‐ FD – (1‐ FD)^*X*^) for various values of *F*,* D*, and *X*. Multiplying the values displayed in the plots by true prevalence, *P*, gives the difference in bias between individual‐based and anonymous estimates of prevalence. When *X *=* *1, the difference in bias is equal to *P*, as (1 ‐ FD ‐ (1 ‐ FD)^*X*^) = 1 for all values of *F* and *D*

## APPENDIX 1

### Calculating Infection Prevalence: Best Practices and Their Theoretical Underpinnings

#### DERIVATION OF EQUATION 3

$$\text{Eqn 3}:\;\;\;E\left[\hat{P}\right]=P\left(1-{\left(1-D\right)}^{X}\right)$$

Equation (3) gives the expected value of an individual‐based estimate of prevalence. *D* is the probability that a sample from an infectious individual will test positive. All samples from infected individuals are assumed to contain the evidence of infection needed to test positive, and to be equally likely to test positive. *P* is the true prevalence.

The probability of a single sample from a single infected individual not being detected as infected is 1‐ *D*. The probability of all samples from the same individual not being detected as infected is (1‐*D*)^*X*^. Therefore, the probability of an infected individual being detected as infected is 1‐ (1‐*D*)^*X*^. The number of individuals expected to be infected in a sample of the population is *P***n*, so the total number of individuals expected to be detected as infected is *Pn*(1 ‐ (1 ‐ *D*)^*X*^). Dividing by the total number of individuals sampled gives the expected estimate of prevalence $\frac{P_{n}\left(1-{\left(1-D\right)}^{X}\right)}{n}$ which reduces to $P(1-{\left(1-D\right)}^{X})$

#### DERIVATION OF EQUATION 4

$$\text{Eqn 4}:\;\;\;E\left[\hat{P}\right]=P\left(1-{\left(1-\text{FD}\right)}^{X}\right)$$

Equation (4) gives the expected value of the estimate of prevalence in the same scenario as in Equation (3), but with the added assumption that samples from an infected individual contain evidence of an infection with probability *F*.

The probability of a single sample from a single infected individual not being detected as infected is the sum of the probability that the sample contains evidence of infection and is not detected as infected (*F* * (1‐*D*)) and the probability that the sample does not contain infectious material and is not detected as infected ((1‐*F*)*1). This sum is equal to 1‐ FD. The probability of all samples from an infected individual not being detected as infected is (1‐FD)^*X*^ and therefore the probability that an infected individual is detected as infected is 1‐ (1‐FD)^*X*^. The number of individuals expected to be detected as infected in a population is $Pn(1-{\left(1-\text{FD}\right)}^{X})$. Dividing by the total number of individuals sampled gives the expected estimate of prevalence $\frac{Pn(1-{\left(1-\mathrm{FD}\right)}^{X})}{n}$ which reduces to $P(1-{\left(1-\text{FD}\right)}^{X})$

#### DERIVATION OF EQUATION 6

$$\text{Eqn 6}:\;\;\;E[\hat{P}]=\text{PDF}$$

Equation (6) gives the expected value of the estimate of prevalence for anonymous estimation. We assume that all individuals generate the same number of samples regardless of their infection status and that samples are randomly selected from the “pool” of all samples. The number of samples generated from infected individuals containing evidence of infection will be *S*_N_ PF. The number of these that will be detected as containing infectious material will be *S*_N_ PFD Therefore, the expected proportion of samples detected as containing infectious material in a population will be $\frac{S_{N}\mathrm{PFD}}{S_{N}}$, which reduces to Equation (6).

#### COMPARISON OF BIASES FOR ANONYMOUS PREVALENCE ESTIMATION AND INDIVIDUAL‐BASED PREVALENCE ESTIMATION

The bias of individual‐based prevalence estimation is P((1‐(1‐FD)^x^)‐1) (equation 4). The bias of anonymous prevalence estimation is P(FD‐1) (equation 6).

If *X *=* *1,

P((1‐(1‐FD)^x^)‐1) = P((1‐(1‐FD)‐1) = P(FD‐1)

Therefore, no difference in bias exists between anonymous estimation and individual‐based estimation with single sampling.$X>1,\;\;\text{FD}<1-{\left(1-\text{FD}\right)}^{X}$

To prove that the bias is always more negative for anonymous sampling than for individual‐based sampling when *X* > 1, we wish to show that *P*(FD − 1) < *P*((1 − (1 − FD)^*X*^)−1).

$$\text{FD}-1\;<\;(1-{\left(1-\text{FD}\right)}^{X})-1$$

$$\begin{array}{cc} & \text{FD}<1-{\left(1-\text{FD}\right)}^{X}\\ & \text{FD}-1<-{\left(1-\text{FD}\right)}^{X}\\ & 1-\text{FD}>{\left(1-\text{FD}\right)}^{X}\\ & 1>\text{FD}>0\Rightarrow 1>1-\text{FD}>0\\ & \Rightarrow \;1-\text{FD}>{\left(1-\text{FD}\right)}^{X} \end{array}$$

#### SENSITIVITY ANALYSIS OF PREVALENCE BIASES

Here, we consider the difference in bias for individual‐based and anonymous prevalence estimation methods applied to the same system (i.e. same values of *F*,* D*, and *P*). The difference in bias is:

$$\begin{array}{cc} & P(\left(1-{\left(1-FD\right)}^{X}\right)-1)-P\left(FD-1\right)\\ & =P(1-{\left(1-\text{FD}\right)}^{X})-\text{PFD}\\ & =P(1-\text{FD}-{\left(1-FD\right)}^{X}) \end{array}$$

Thus, the difference in bias is expected to be directly proportional to true prevalence, *P*. Figure A1 above shows that in addition to increasing with *P*, the difference in bias between the two methods increases with greater values of *X* and is maximized at different combinations of *F* and *D*, depending on the value of *X*.
